# Combined Use of Whole Exome Sequencing and CRISPR/Cas9 to Study the Etiology of Non-Obstructive Azoospermia: Demonstration of the Dispensable Role of the Testis-Specific Genes *C1orf185* and *CCT6B*

**DOI:** 10.3390/cells11010118

**Published:** 2021-12-30

**Authors:** Caroline Cazin, Yasmine Neirijnck, Corinne Loeuillet, Lydia Wehrli, Françoise Kühne, Isabelle Lordey, Selima Fourati Ben Mustapha, Amin Bouker, Raoudha Zouari, Nicolas Thierry-Mieg, Serge Nef, Christophe Arnoult, Pierre F. Ray, Zine-Eddine Kherraf

**Affiliations:** 1 Team Genetics Epigenetics and Therapies of Infertility, Institute for Advanced Biosciences, University Grenoble Alpes, INSERM U1209, CNRS UMR 5309, 38000 Grenoble, France; EXT-CCazin@chu-grenoble.fr (C.C.); corinne.loeuillet@univ-grenoble-alpes.fr (C.L.); christophe.arnoult@univ-grenoble-alpes.fr (C.A.); PRay@chu-grenoble.fr (P.F.R.); 2 UM GI-DPI, CHU Grenoble Alpes, 38000 Grenoble, France; ilordey@chu-grenoble.fr; 3 Department of Genetic Medicine and Development, Faculty of Medicine, University of Geneva, CH-1211 Genève 4, Switzerland; Yasmine.NEIRIJNCK@univ-cotedazur.fr (Y.N.); Lydia.Wehrli@unige.ch (L.W.); Francoise.Kuhne@unige.ch (F.K.); Serge.Nef@unige.ch (S.N.); 4 Centre d’Aide Médicale à la Procréation, Polyclinique les Jasmins, Centre Urbain Nord, Tunis 1003, Tunisia; fourati_selima@yahoo.fr (S.F.B.M.); aminbouker@gmail.com (A.B.); raoudha.zouari@cliniquelesjasmins.com.tn (R.Z.); 5 TIMC-IMAG, CNRS and Université Grenoble Alpes, 38000 Grenoble, France; Nicolas.Thierry-Mieg@univ-grenoble-alpes.fr

**Keywords:** spermatogenesis, non-obstructive azoospermia, genetics of male infertility, whole exome sequencing, CRISPR/Cas9, *C1orf185*, *CCT6B*

## Abstract

The genetic landscape of male infertility is highly complex. It is estimated that at least 4000 genes are involved in human spermatogenesis, but only few have so far been extensively studied. In this study, we investigated by whole exome sequencing two cases of idiopathic non-obstructive azoospermia (NOA) due to severe hypospermatogenesis. After variant filtering and prioritizing, we retained for each patient a homozygous loss-of-function (LoF) variant in a testis-specific gene, *C1orf185* (c.250C>T; p.Gln84Ter) and *CCT6B* (c.615-2A>G), respectively. Both variants are rare according to the gnomAD database and absent from our local control cohort (*n* = 445). To verify the implication of these candidate genes in NOA, we used the CRISPR/Cas9 system to invalidate the mouse orthologs *4930522H14Rik* and *Cct6b* and produced two knockout (KO) mouse lines. Sperm and testis parameters of homozygous KO adult male mice were analyzed and compared with those of wild-type animals. We showed that homozygous KO males were fertile and displayed normal sperm parameters and a functional spermatogenesis. Overall, these results demonstrate that not all genes highly and specifically expressed in the testes are essential for spermatogenesis, and in particular, we conclude that bi-allelic variants of *C1orf185* and *CCT6B* are most likely not to be involved in NOA and male fertility.

## 1. Introduction

Infertility, or the inability to conceive, remains a prevalent and ongoing global health concern [1]. Infertility is currently estimated to affect 9% of reproductive-aged couples worldwide, with approximately half involving a male factor [2,3]. The male factor is related to a variety of causes ranging from genetic abnormalities to lifestyle choice [4]. The genetic landscape of male infertility is highly complex as semen and testis histological phenotypes are extremely heterogeneous. It is estimated that at least 4000 genes are involved in human spermatogenesis, but only few have so far been extensively studied [5].

A comprehensive and in-depth understanding of genetic factors in spermatogenesis abnormalities will play important roles in the clinical diagnosis, treatment, and genetic counseling of male infertility. Currently, the discovery of novel genetic factors in idiopathic infertility is a major challenge in the fields of reproductive medicine and andrology. The highest frequency of known genetic factors contributing to male infertility (25%) is in azoospermia [6]. The diagnostic yield of genetic tests in azoospermia varies depending on the etiology of the disorder, with a diagnosis yield of approximately 90% for congenital bilateral absence of vas deferens (obstructive azoospermia) and only 30% due to a spermatogenic defect (NOA) [7]. This last condition is known as non-obstructive azoospermia (NOA) and is considered as the most severe form of male infertility. In addition, non-obstructive azoospermic men showed the worst health status impairment and should be strictly followed-up regardless of their fertility status [8].

The use of whole exome sequencing (WES) for the detection of disease-causing variants of genetic diseases is a major clinical application of next generation sequencing (NGS). WES allowed the discovery of an increasing number of monogenic defects of NOA with a current list of 38 candidate genes [7]. However, a major difficulty remains as to how to proceed with the many candidates with a possible but unconfirmed pathogenic effect (variants of unknown significance, VUS) that remains after filtering with all available methods. Functional analyses, especially for genes that are not yet well characterized, can be time-consuming and expensive [9]. 

The mouse model, due to its amenability and its genetic and physiological similarities with man, is extensively used to study human health and diseases. The creation of genetically modified mice as models of human disease has remarkably changed our ability to understand the molecular mechanisms and cellular pathways underlying disease states. Knockout (KO) mice have been pivotal in studying gene function in vivo. Historically, the study of KO animals is the “gold standard” to determine whether a gene’s function is essential in vivo. In the era of CRISPR/Cas9-based genome engineering, the generation of genetically modified mice becomes much more flexible, accurate, efficient, and cost-effective. The functions of testis-specific genes can be assessed in vivo by creating male mice carrying a null mutation in the genes of interest. Upon the production of KO mouse lines, the essentiality of the deleted genes can be readily determined by examining the fertility of homozygous male offspring.

In the present study, we investigated by WES two unrelated infertile patients born from consanguineous parents and displaying idiopathic non-obstructive azoospermia associated with severe hypospermatogenesis. After variants filtering and prioritizing, we retained for each individual a homozygous truncating variant in a testis-specific gene (*C1orf185 or CCT6B*). To study the impact of loss-of-function (LoF) bi-allelic variants of these genes in sperm production and male fertility, we generated two KO mouse lines by introducing homozygous frameshift mutations in the corresponding orthologs *4930522H14Rik* and *Cct6b*. Homozygous KO males were fertile and showed normal sperm production, thus excluding an essential role of these genes for spermatogenesis and male fertility. 

Overall, these results demonstrate the primordial role of in vivo testing of the impact of candidate testis-specific genes on spermatogenesis and male fertility through the production of KO mouse lines after WES investigation of infertile patients and the identification of candidate LoF variants. The current and future efforts to examine the molecular and genetic factors responsible for spermatogenesis and fertilization should give a better understanding of the etiologies of male infertility and enable improves in patient care [4]. 

## 2. Material and Methods

### 2.1. Patients and Biological Samples

Two unrelated infertile men were included in the present study displaying non-syndromic infertility due to idiopathic non-obstructive azoospermia. Subjects were recruited at the Clinique des Jasmins in Tunis (Tunisia). 

Informed consent was obtained from all individuals participating in the study according to local protocols and the principles of the Declaration of Helsinki. The study was approved by local ethics committees, and samples were then stored at the Fertithèque collection declared to the French Ministry of Health (DC-2015–2580) and the French Data Protection Authority (DR-2016–392).

### 2.2. WES and Variant Filtering

Genomic DNA was isolated from blood samples or saliva using the Oragen DNA Extraction Kit (DNA Genotek Inc., Ottawa, ON, Canada). Genetic data were obtained from Novogene (Hong Kong, China, HiSeqX). Coding regions and intron/exon boundaries were sequenced after enrichment using SureSelect Human All Exon V6 from Agilent.

An alignment-ready GRCh38 reference genome (including alternative sequences (ALT), decoy, and human leukocyte antigens (HLA)) was produced using “run-gen-ref hs38DH” from Heng Li’s bwakit package [10]. The exomes were analyzed using a bioinformatics pipeline developed in-house. The pipeline consists of two modules, both distributed under the GNU General Public License v3.0 and available on github.

The first module (https://github.com/ntm/grexome-TIMC-Primary, accessed on 5 November 2018) takes FASTQ files as input and produces a single merged GVCF file, as follows. Adaptors are trimmed and low-quality reads are filtered with fastp 0.20.0 [11], reads are aligned with BWA-MEM 0.7.17 [12], duplicates are marked using samblaster 0.1.24 [13], and BAM files are sorted and indexed with samtools 1.9 [14]. SNVs (single nucleotide variants) and short indels are called from each BAM file using strelka 2.9.10 to produce individual GVCF files [15]. These are finally merged with GVCFs.pl to obtain a single multi-sample GVCF, which combines all exomes available in our laboratory.

The second module (https://github.com/ntm/grexome-TIMC-Secondary, accessed on 20 November 2018) takes this merged GVCF as input and produces annotated analysis ready TSV files. This is achieved by performing up to 15 streamlined tasks including the following: low-quality variant calls (read depth (DP) < 10, genotype quality for variant filtration (GQX) < 20, or less than 15% of reads supporting the ALT allele) are discarded; Variant Effect Predictor v92 [16] is used to annotate the variants and predict their impact, allowing us to filter low-impact variants and/or prioritize high-impact ones (e.g., stop-gain or frameshift variants) [16]; gene expression data from the Genotype-Tissue Expression project (GTEx v7) are added; variants with a minor allele frequency greater than 1% in gnomAD v2.0, 3% in 1000 Genomes Project phase 3, or 5% in NHLBI (National Heart, Lung, and Blood Institute) ESP6500 are filtered. Variants are also compared to those obtained from 485 exomes of healthy control individuals or of patients presenting a clearly different phenotype. Because all variants result from the same bioinformatics pipeline, this allows us to filter artifacts due to the pipeline itself as well as genuine variants that may be missing from public databases, but are actually not so rare in our cohorts. Finally, the resulting TSV files can be opened with spreadsheet software such as LibreOffice Calc or Microsoft Excel for further filtering and sorting, in order to identify candidate causal variants.

### 2.3. Sanger Verification of the Variant

Variants identified by WES were subjected to Sanger verification using an ABI 3500XL Genetic Analyzer (Thermo Fisher Scientific, Waltham, MA, USA). Analyses were performed using SeqScape software 3.0 (Applied Biosystems, Foster City, CA, USA). Primer sequences and their expected product sizes are summarized in Supplementary Table S3.

### 2.4. CRISPR/Cas9—Mediated Mice Genome Edition

CrispR/Cas9 gene editing was used to knockout *4930522H14Rik* and *Cct6b*, respectively, as described in Supplementary Figure S1A. To produce a large frameshift deletion, we designed a dual-gRNA targeting a single coding exon at the beginning of the gene of interest (Supplementary Table S4). Guide RNA, TracRNA, ssDNA, and Cas9 were purchased from Integrated DNA Technologies. Oocyte injection and embryo transfer were performed by the Transgenic Core Facility of the Faculty of Medicine, University of Geneva. Briefly, gRNA and TracRNA were annealed at equimolar concentration prior to complex formation with the Cas9 nuclease. Ribonucleoprotein complexes were co-injected into B6D2F1 oocytes. Microinjected oocytes were introduced into pseudopregnant host females and carried to term. Edited founders were identified by PCR and Sanger sequencing from digit biopsies. Mice carrying the desired modification events (frameshift mutation) were crossed with B6D2F1 to ensure germline transmission and eliminate any possible mosaicism. Heterozygous animals with the same modification were then mated to generate homozygous offspring (Supplementary Figure S1B).

### 2.5. Mice Genotyping Strategy

DNA for genotyping was isolated from tail biopsies. Tail biopsies (2 mm in length) were digested in 200 µL of Direct PCR Lysis Reagent (Tail) (Viagen Biotech Inc, Los Angeles, CA, USA) and 0.2 mg of proteinase K for 12–15 h at 55 °C followed by 1 h at 85 °C for proteinase K in activation. The DNA was directly used for PCRs. PCR products were separated by 2% agarose gel electrophoresis. Genotypes were determined according to the migration pattern. Primers are described in Supplementary Table S5. Sequence analyses were carried out on ABI3500XL (Applied Biosystems). Sequences were analyzed using seqscape software (Applied Biosystems).

### 2.6. Phenotypic Analysis of Mutant Mice

All procedures were conducted in Geneva until the birth of the modified litters (F0 generation). Animals of interest were then transferred to the University Grenoble Alpes (UGA). All animal work was conducted according to the ethical guidelines of the French local Ethical Committee (ComEth Grenoble No. 318, Ministry Agreement Number #7128 UHTA-U1209-CA) and the Direction Générale de la Santé (DGS) for the State of Geneva.

Mice were housed with unlimited access to food and water and were sacrificed by cervical dislocation after they were eight weeks old, which means that they were pubescent and that their reproductive organs were fully established. 

To test fertility, pubescent homozygous KO and WT males (8 week-old) were mated with WT females for at least two months. The females were checked for the presence of vaginal plugs and pregnancy. The number of mice achieving a pregnancy and the litter size of each mating set or pregnancy were recorded. We tested in this study (due to the COVID-19 pandemic) a single KO animal and at least two WT animals for each mouse line. Because the main aim of this study was to validate or invalidate the implication of the identified candidate genes in NOA, we focused our phenotypic analysis on quantitative spermatogenic defects in KO mice, leading to oligozoospermia or azoospermia. The qualitative defects such as morphological and functional defects of sperm cells produced by KO animals were not fully investigated in this study, and therefore related data are not reported in the present paper.

To determine sperm concentration, adult males were euthanized and sperm samples were collected from the cauda epididymis and vas deferens. Spermatozoa were released in 1 mL of PBS solution by making a series of incisions in these ducts. Sperm number was determined using a hemocytometer under a light microscope, after 2 min of fixation by paraformaldehyde 4% to immobilize motile cells. Sperm motility and morphology were examined under a light microscope to rule out any obvious abnormalities compared to WT animals.

To analyze testicular integrity, testes from adult wild-type (WT) and KO mice were fixed by immersion in 4% paraformaldehyde (PFA) for 14 h, embedded in paraffin, and sectioned (7 µm). For histological analysis, after being deparaffinized, slides were stained with hematoxylin and eosin. The colored sections were digitized at ×20 magnification through an axioscan slide scanner (Zeiss, Germany) equipped with a motorized X–Y-sensitive stage. 

### 2.7. Statistical Analyses

n represents the number of biological replicates. For sperm analyses, for each replicate, more than 100 sperm were assessed per condition. Statistical analyses were performed with GraphPAD prism software 6 (San Diego, CA, USA). *t*-Tests were used to compare the WT and KO samples. Data represent mean ± SEM or SD, as indicated. Statistical tests with a two-tailed *p*-value ≤ 0.05 were considered significant.

## 3. Results

### 3.1. Medical Assessment of Two Infertile Men Displaying Idiopathic Non-Obstructive Azoospermia

**Figure 1:**
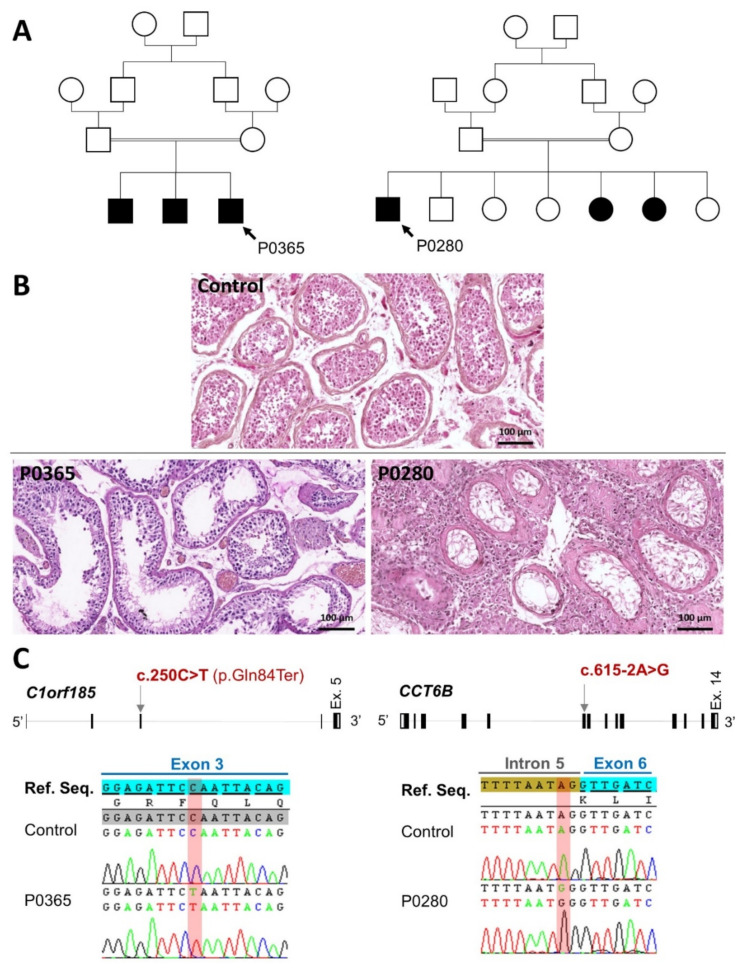
Genetic investigation of two idiopathic cases of non-obstructive azoospermia (NOA) and functional assessment of variant pathogenesis. (**A**) Pedigrees of the two studied subjects P0365 and P0280. Black color indicates individuals with primary infertility. (**B**) Histology of testicular seminiferous tubules obtained from the studied subjects after multifocal testicular biopsies showing a severe hypospermatogenesis in subject P0365 and a testicular degeneration in subject P0280 compared to the control (upper panel). Scale bars = 100 µm. (**C**) Location of the identified variants in the candidate genes and the corresponding electropherograms obtained by Sanger sequencing. P0365 has a homozygous stop-gained variant in C1orf185 (c.250C>T; p.Gln84Ter) whereas P0280 carries a homozygous variant affecting a splice acceptor consensus sequence in CCT6B (c.615-2A>G).

Two unrelated men, P0280 and P0365, with an age of 44 and 43 years, respectively, sought medical care at the Clinique des Jasmins in Tunis (Tunisia) for primary infertility. Both patients were of North African origin and were born from related parents (Figure 1A). Analyses of their ejaculates evidenced a total absence of sperm cells (Table 1). P0280 presented very small testes (<5 mL) with elevated follicular stimulating hormone (FSH) level (37 UI/L normal range: 1.5–12.4) whereas P0365 had a normal FSH level (5.73 UI/l) and a testis volume ranging between 10–15 mL (normal > 15 mL). Patients did not present any symptom related to hypogonadism. Plasmatic testosterone level in these subjects was normal, ranging from 3.45 to 7.3 ng/mL. Karyotypes were normal (46,XY) and Y chromosome microdeletion within the AZF (azoospermia factor) region was excluded.

Men displaying NOA may have sperm cells in their testes that could be retrieved through a surgical procedure such as microdissection testicular sperm extraction (mTESE) [17]. mTESE was performed in both patients for assisted reproduction. Histological analysis of the testicular fragments evidenced a severe quantitative defect of spermatogenesis (Figure 1B). P0365, a sub-testicular phenotype of hypospermatogenesis characterized by the rarefaction of germ cells within the seminiferous tubules, leads to an extremely low production of elongated spermatids. The mTESE procedure was positive in this case and allowed us to retrieve few sperm cells with bad quality. For P0280, the testicular sub-phenotype was more severe compared to P0365 and evidenced severe hypospermatogenesis associated with seminiferous tubules hyalinization. As expected for this case, mTESE failed to retrieve spermatozoa.

### 3.2. WES and Variant Filtering

We used a WES-based strategy to investigate two patients in order to identify the potential genetic origin responsible for their infertility. Given the familial history of consanguinity, we postulated that these genetic factors had a likely autosomal recessive inheritance. Therefore, we focused our analyses on homozygous variants with an allelic frequency (AF) <1%. After variant filtering and prioritizing, we selected and retained for each patient a homozygous truncating variant in *C1orf185* or *CCT6B.* All the identified variants were rare (AF = 5.02 × 10^−3^ and 3.53 × 10^−3^, respectively) according to the gnomAD database and were absent from our local control cohort (*n* = 445). These candidate genes were predicted, according to in silico databases, to be highly and specifically expressed in testes contrasting with an unknown biological function. We also looked for other genes with another tissue expression pattern and did not find any relevant candidate variants. We paid particular attention to genes with high expression in both male and female gonads when analyzing the exome data from P0280. The familial history of this subject showed that he has two infertile sisters. This information could orientate us toward a common genetic cause that explains the infertility of these siblings, particularly if the women display primary ovarian insufficiency, a comparable phenotype with NOA in men. However, these cases have not been documented and in addition, we were unable to obtain their DNA samples to perform genetic segregation analyses.

*C1orf185* (chromosome 1 open reading frame 185) is located on chromosome 1 (1p32.3). The canonical transcript (ENST00000371759.7; NM_001136508.2) contains five exons coding for a protein of 199 residues. We identified a nonsense variant (c.250C>T; p.Gln84Ter) located in exon 3 and was suspected to either produce a truncated protein or to induce the degradation of the mRNA by the nonsense-mediated mRNA decay (NMD). *CCT6B* (chaperonin containing TCP1 subunit 6B) is located on chromosome 17 (17q12). The canonical transcript (ENST00000314144.10; NM_006584.4) contains 14 exons coding for a protein of 530 residues. We identified in this gene a splicing variant (c.615-2A>G) affecting the second nucleotide of the splice acceptor consensus sequence located in intron 5 (Figure 1C). The homozygous state of these variants was confirmed by Sanger sequencing (Figure 1C). In conclusion, we identified two homozygous loss-of-function variants in two testis-specific genes with an unknown function that appear to be good candidates to explain the infertility and the spermatic phenotype observed in our patients.

### 3.3. Generation of KO Mice by CRISPR/Cas9 System

To study the impact of the identified candidate variants in *C1orf185* and *CCT6B,* we targeted the corresponding mouse orthologs *4930522H14Rik* and *Cct6b*, respectively, using the CRISPR/Cas9 system. Before starting this procedure, we compared and aligned the DNA and protein sequences from each ortholog to confirm their homology (Supplementary Tables S1 and S2). We also performed a phylogenetic analysis on protein sequences to understand the evolution of these genes among species and compared the tissue expression profile between the orthologs in human and mice (Supplementary Figures S2–S5). We showed that mouse genes, similar to their human orthologs, are specifically expressed in the testes (Figures S3 and S5). We did not identify a paralogue for *C1orf185.* However, we found a paralog of *CCT6B* named *CCT6A* that has a distinct ortholog in mice, *Cct6a*.

We then designed for each gene two guide RNAs (gRNAs) targeting two coding sequences located in a single exon to produce a large intra-exonic frameshift deletion. The Cas9 protein and the dual-gRNAs were injected during ICSI procedure in mature oocytes. We then transferred embryos in pseudopregnant females and obtained 21 and 34 pups (F0 generation) after targeting *4930522H14Rik* and *Cct6b,* respectively. The allelic mutational events were counted to estimate the efficiency of our strategy. We obtained a mutational rate of 100% by targeting *4930522H14Rik* and *Cct6b*. We then selected a frameshift large intragenic deletion and backcrossed mutated females (suspected fertile) from F0 generation with WT males to obtain heterozygous mice (F1 generation). F1 heterozygous males were then mated with heterozygous females carrying the same mutation to obtain homozygous mutated mice (F2 generation).

### 3.4. Phenotypic Analysis of Adult KO Male Mice

Phenotypic analyses were carried out for the homozygous KO male mice in parallel with the same-aged WT controls from the F2 generation to investigate spermatic and testicular phenotypes. In addition, we tested the fertility of homozygous KO animals. No abnormal somatic development or behavior was observed in any of the KO mouse lines generated in this study.

**Figure 2:**
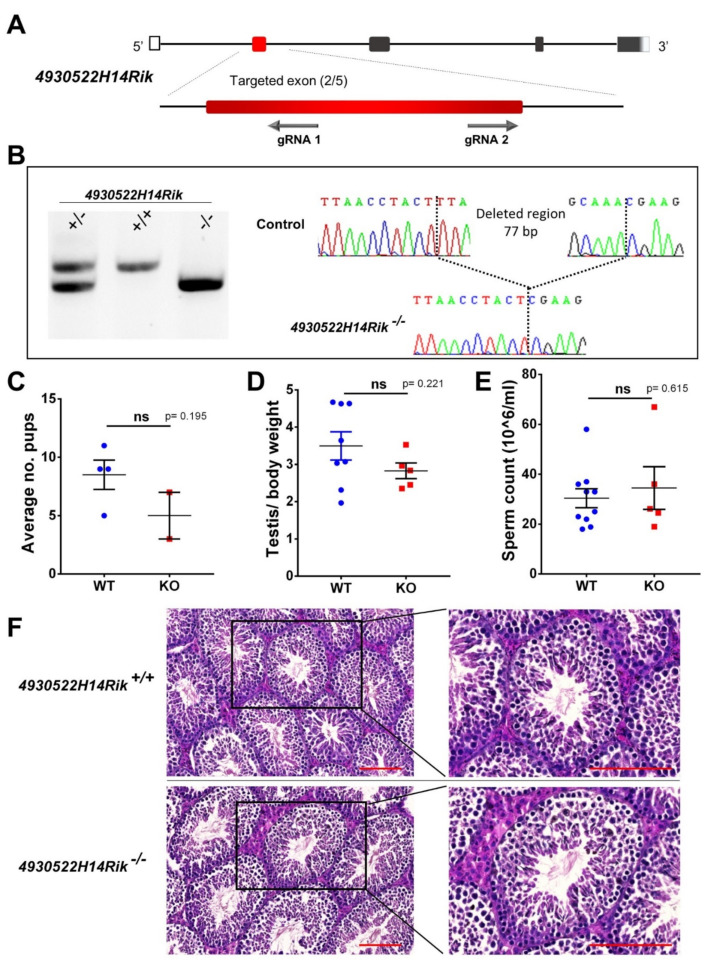
*4930522H14Rik* editing, genotyping strategy, and phenotypic analyses of mutated animals. (**A**) Schematic presentation of 4930522H14Rik highlighting the targeted exon in red (exon 2/5) and showing the approximate location of the sequences targeted by the dual gRNAs. (**B**) Gel electrophoresis of the PCR products using a primer set that amplifies the region containing the intra-exonic deletion. DNA fragments migrate according to their molecular size (WT allele: 457 bp and KO allele: 380 bp). Genotyping strategy was completed by Sanger sequencing of PCR products for F0–F1 generations. (**C**) Average litter size of WT and KO male mice. (**D**) Comparison of testis to body weight ratios between WT (*n* = 8) and KO (*n* = 5) animals. (**E**) Comparison of sperm count between WT (*n* = 10) and KO (*n* = 5) animals. (**F**) Histological analyses of WT and KO testicular sections after hematoxylin and eosin staining. Scale bar = 100 μm.

The *4930522H14Rik* (Ortholog of *C1orf185*) KO line was generated using two gRNAs designed to target two coding sequences located in exon 2 (Figure 2A). Genotyping was performed using PCR followed by gel electrophoresis and Sanger sequencing of the PCR products (Figure 2B). We selected a 77 bp frameshift deletion (c.41-117del) to generate the KO line (Figure 2B). Homozygous KO males sired pups of comparable litter size as WT (*p*-value = 0.195) (Figure 2C). We performed morphological and histological analysis of the testes and showed no significant differences in appearance and testis to body weight ratio (*p*-value = 0.221) (Figure 2D). We collected and analyzed the content of the cauda epididymis and showed that homozygous adult KO males displayed a normal sperm count (Figure 2E) (data not shown) without a significant difference compared to the control animals (*p*-value = 0.615). Histological analysis of testicular section from KO and WT animals showed normal architecture of the testicular parenchyma (Figure 2F). We analyzed numerous sections of seminiferous tubules and observed a functional spermatogenesis in KO compared to WT (Figure 2F).

**Figure 3:**
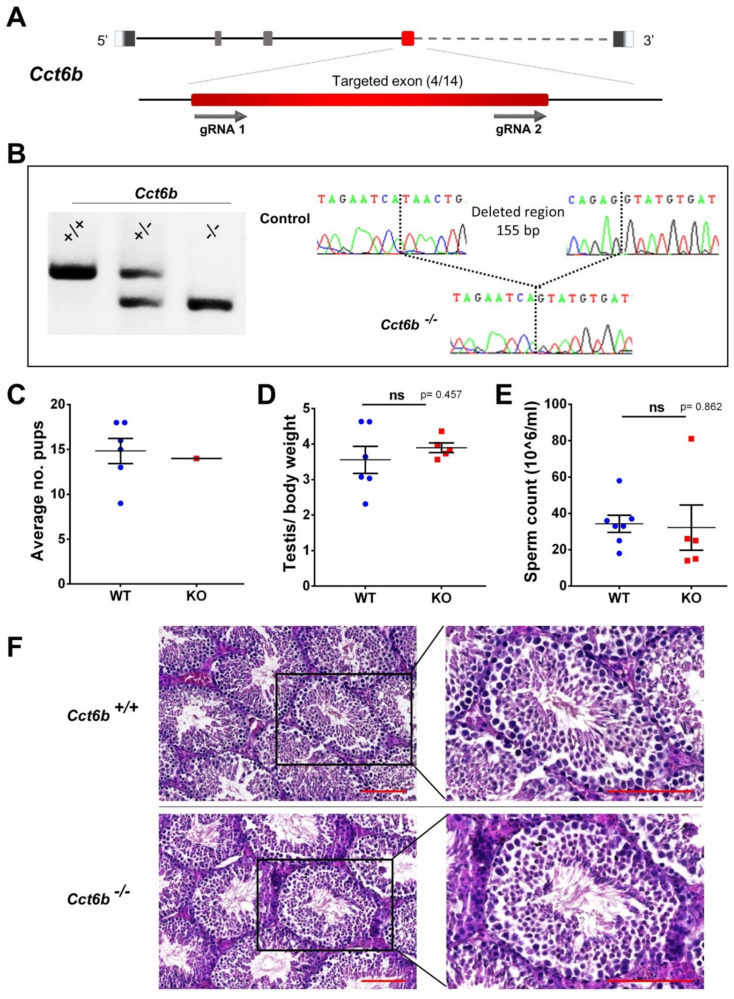
*Cct6b* editing, genotyping strategy, and phenotypic analyses of mutated animals. (**A**) Schematic presentation of Cct6b highlighting the targeted exon in red (exon 4/14) and showing the approximate location of the sequences targeted by the dual gRNAs. (**B**) Gel electrophoreses of the PCR products using a primer set that amplifies the region containing the intra-exonic deletion. DNA fragments migrate according to their molecular size (WT allele: 535 bp and KO allele: 380 bp). Genotyping strategy was completed by Sanger sequencing of PCR products for F0–F1 generations. (**C**) Litter size of WT and KO males (a single litter was recorded due to a short mating period). (**D**) Comparison of testis to body weight ratios between WT (*n* = 6) and KO (*n* = 5) animals. (**E**) Comparison of sperm count between WT (*n* = 7) and KO (*n* = 5) animals. (**F**) Histological analyses of WT and KO testicular sections after hematoxylin and eosin staining. Scale bars = 100 μm.

To obtain the *Cct6b* KO mouse line, we generated two gRNAs targeted two coding sequences within exon 4 (Figure 3A). We performed PCR followed by gel electrophoresis and Sanger sequencing of the PCR products in order to genotype mice (Figure 3B). A 155 bp frameshift deletion (c.354_505del) was chosen to create the KO line (Figure 3B). Litter size from homozygous KO males was comparable to the control (Figure 3C). Statistical analyses could not be performed for this test because we obtained a single litter from KO males before euthanizing the mouse line due to the restrictions on the use of animal facilities during the first wave of the COVID pandemic. Otherwise, we can conclude that *Cct6b* KO males were not sterile. Then, morphological and histological analysis of testes showed no significant differences in appearance and testis to body weight ratio (*p*-value = 0.457) (Figure 3D). After extraction and analysis of sperm from the cauda epididymis, we showed that homozygous adult KO males had a normal sperm count (Figure 3E) and (data not shown) without significant difference from WT males (*p*-value = 0.862). Histological analysis of testicular sections from KO and WT animals showed that they presented similar parameters with normal parenchyma architecture and functional spermatogenesis (Figure 3F).

## 4. Discussion

Currently, the discovery of new candidate genes and causal variants in idiopathic male infertility is a major challenge in the fields of andrology and reproductive medicine. The recent development of high throughput sequencing (HTS) techniques and the availability of WES for research and clinical practice allowed for the discovery of several novel candidate genes responsible for spermatogenic defects and male infertility [18,19,20]. Non-obstructive azoospermia (NOA) is a frequent and very severe cause of male infertility characterized by a strong genetic basis. Taking into account the high number of genes predominantly or specifically expressed during spermatogenesis and involved in biological processes such as mitosis, meiosis, cell differentiation, genome stability, and retro-element neutralization, NOA is expected to be highly heterogeneous and could be mainly caused by monogenic mutations [21]. In 2018, Fakhro et al. investigated by WES a cohort of 75 unrelated subjects displaying idiopathic NOA and identified monogenic causes in 10 cases (13%) [22]. In 2020, Chen et al. investigated by WES a large cohort of 314 infertile subjects presenting NOA or severe oligospermia and identified 20 novel candidate genes affecting 25 patients [23]. During the same year, Krausz et al. published a paper reporting the identification of five novel NOA candidate genes by testing 147 selected patients displaying a spermatogenic arrest [24].

Despite the robustness of WES to study the genetic component of idiopathic NOA, many deleterious variants in genes with uncharacterized biological function are classified as variants of unknown clinical significance (VUS) [25]. Although gene-expression analysis reveals the presence of thousands of testis-enriched genes, the biological function of most of these genes remain unknown [26,27]. In this study, we reported the identification of homozygous LoF variants in two testis-enriched and specific genes of unknown function *C1orf185* (c.250C>T; p.Gln84Ter) and *CCT6B* (c.615-2A>G). To assess the implication of these candidate genes in NOA and male fertility, we invalidated their mouse orthologs using the CRSIPR/Cas9 system and generated two KO lines. The same strategy has previously allowed us to gain a better understanding of the genetic etiology of several spermatogenic defects such as NOA and monomorphic teratozoospermia [28,29,30].

Using CRSIPR/Cas9 technology, gene edition in mice is now relatively easy and fast, allowing for the generation of several knockout and/or knockin lines in only a few months [31]. The method used in this study consisted of using two gRNAs with a distance of approximately 100 bp from each other and targeting a single exon in the gene of interest. The dual-gRNAs and the Cas9 protein were injected into mature oocytes during the ICSI procedure. Experimenting with this new strategy, we significantly increased the efficiency of this system and generated sufficiently large frameshift deletions in pups of generation F0, allowing us to establish KO lines by performing a single cycle of oocyte injection. In addition, the introduction of large intra-exonic deletions of approximately 100 bp allows for fast genotyping. Indeed, the use of a single set of primers in PCR allows for the amplification in the same reaction of the mutated and the WT alleles, which can be easily differentiated after a simple gel electrophoresis. Using this strategy, we performed PCR and Sanger sequencing to genotype pups from F0 and F1 generations and only PCR for further genotyping, thus facilitating the management of mouse colonies.

In this study, we focused our phenotypic analyses to study the reproductive system and demonstrate whether adult male mice exhibited an altered spermatogenesis. We performed a fertility test by mating homozygous KO males with WT females and observing the litter size following at least two months. We observed that *4930522H14Rik^−/−^* and *Cct6b^−/−^* males displayed normal fertility compared to WT males born from the same parents. We also showed that KO males from both lines presented normal sperm parameters and spermatogenesis compared to control littermates.

In June 2021 and during the preparation of our manuscript, Yang et al. published an interesting paper reporting the reproductive phenotype of *Cct6b^−/−^* mice generated by the CRISPR/Cas9 system by targeting the same exon (4/14). Concordant with what we describe here, they found that males exhibited no differences in development, fertility, testis weight, sperm counts, total motility, and spermatogenesis relative to control littermates, thus confirming our results [32].

During the past five years, many teams searching for target proteins for non-hormonal male contraceptive drugs published their studies reporting the dispensable role of numerous testis-enriched genes in spermatogenesis and male fertility through a massive production of KO mice using the CRISPR/Cas9 system [26,33,34,35,36]. Reporting genetic variants and genes non-associated with male infertility individually is important and necessary to avoid the further creation of these KO mouse lines and the duplication of efforts and to avoid loss of time and significant expenses.

## 5. Conclusions

Whole exome sequencing (WES) is a robust and one of the most comprehensive genetic tests to identify rare disease causing variants and new candidate genes in a wide variety of spermatogenic defects associated with male infertility. Functional interpretation performed in candidate genes is now a critical step to validate their involvement in the studied phenotype. Among the available approaches nowadays, generation of transgenic animal models presents a powerful tool to study the function of candidate genes and their physiopathological implication. The recent development of the CRISPR/Cas9 system has made this process easier and faster, thus combining the use of WES to identify candidate genes and CRISPR/Cas9 to generate knockout mice is a very promising strategy to improve the genetic diagnosis yield and refine the interpretation of variants found in new candidate genes in male infertility.

## Figures and Tables

**Table 1:** Clinical and biological characteristics of the studied subjects.

	P0365	P0280
Age (years)	43	44
Geographical origin	Tunisia	Tunisia
Consanguinity	Yes (1st degree)	Yes (1st degree)
Testosterone (ng/mL) (N: 2.5–10.6)	5.52	3.45
FSH (UI/l) (N: 1.5–12.4)	5.73	37
Karyotype	46,XY	46,XY
AZF microdeletions	Negative	Negative
Testicular volume (left/right, mL) (N: >15)	10–15/10–15	<5/<5
Testicular histology	Severe hypospermatogenesis	Severe hypospermatogenesis associated with seminiferous tubules hyalinization
Sperm retrieval	Positive (rare spermatozoa)	Negative

N = normal range.

## Data Availability

Not applicable.

## Supplementary Materials

The following are available online at https://www.mdpi.com/article/10.3390/cells11010118/s1. Table S1: Comparison between orthologous genes in human and mouse (gene structure, Transcript and protein products; Table S2: Alignment of orthologs DNA and protein sequences; Table S3: Set of primers used for Sanger verification of the variant identified by WES in NOA subjects; Table S4: Characteristics of gRNAs used for CRISPR/Cas9 mouse gene edition; Table S5: Set of primers used for mouse line genotyping; Figure S1: Gene editing using CRISPR/Cas9 system to generate knockout (KO) mouse lines; Figure S2: Tissue expression pattern of C1orf185 and its mouse ortgholog 493022H14Rik; Figure S3: phylogenetic tree of C1orf185 showing their orthologs and paralogs (if present); Figure S4: Tissue expression pattern of CCT6B and its mouse ortolog Cct6b; Figure S5: phylogenetic tree of CCT6b showing their orthologs and paralogs (if present).
