# Supplementary material for: Combined Use of Whole Exome Sequencing and CRISPR/Cas9 to Study the Etiology of Non-Obstructive Azoospermia: Demonstration of the Dispensable Role of the Testis-Specific Genes C1orf185 and CCT6B
Source: Cells. 2021 Dec 30;11(1):118. doi: 10.3390/cells11010118 (PMC8750304; doi:10.3390/cells11010118)
Supplement: Supplementary file 1 [file cells-11-00118-s001.zip › cells-1533466-supplementary.pdf]

SUPPLEMENTARY MATERIALS

SUPPLEMENTARY Tables

Supplementary Table S1. Comparison between orthologous genes in human and mouse (gene structure, Transcript and protein products).

Gene	Exons	Coding exons	Transcript length (bp)	Translation length (a. a.)
C1orf185	5	5	921	199
4930522H14Rik	5	5	854	230
CCT6B	14	14	1847	530
Cct6b	14	14	1787	531

Supplementary Table S2. Alignment of orthologs DNA and protein sequences.

Human/mouse genes	Coding sequence alignment		Protein sequence alignment	
	Query cover	Percent identity	Query cover	Percent identity
C1orf185/ 4930522H14Rik	82 %	73,44	86 %	55,94
CCT6B/Cct6b	98 %	84,79	98 %	84,12

Supplementary Table S3. Set of primers used for Sanger verification of the variant identified by WES in NOA subjects.

Primer name	Primer sequence	Primer Tm (°C)	Product size (bp)
C1orf185_F	AAAGCAATTGATGAGAGATGCAGG	59.90	350
C1orf185_R	CAATCCCAATATGTGGAATGCAGG	60.26	
CCT6B_F	ACAATGTGTGTCCTGGAAGTATTG	59.24	356
CCT6B _R	AAACCAGCTAGTAAAGGCAGGAG	60.31	

Supplementary Table S4. Characteristics of gRNAs used for CRISPR/Cas9 mouse gene edition.

Target gene	Target DNA strand	Target sequence	PAM sequence	On-target score	Off-target score
4930522H14Rik	-	AGCTCCAGCAGCCATAAAGT	AGG	57,5	40,8
	+	GGTTCCTGATTTGCAAACGA	AGG	66,0	43,6
Cct6b	+	CACCCTAGAATCATAACTGA	AGG	61,4	40,3
	+	ACTGGCCGACATACTAACAG	AGG	77,6	44,1

SUPPLEMENTARY MATERIALS

Supplementary Table S5. Set of primers used for mouse line genotyping.

Primer name	Primer sequence	Primer Tm (°C)	Product size (bp)
4930522H14Rik-F	TGTGTCCTGTGTTGCTCTCC	59.89	457
4930522H14Rik-R	GTGGATTGCTTCCTCTCCCC	60.11	
Cct6b-F	ATGTTAGTGCCGTTGCTGTG	59.13	535
Cct6b-R	AGGTGGCATTGGATCCCTTG	60.03	

SUPPLEMENTARY MATERIALS

SUPPLEMENTARY FIGURES

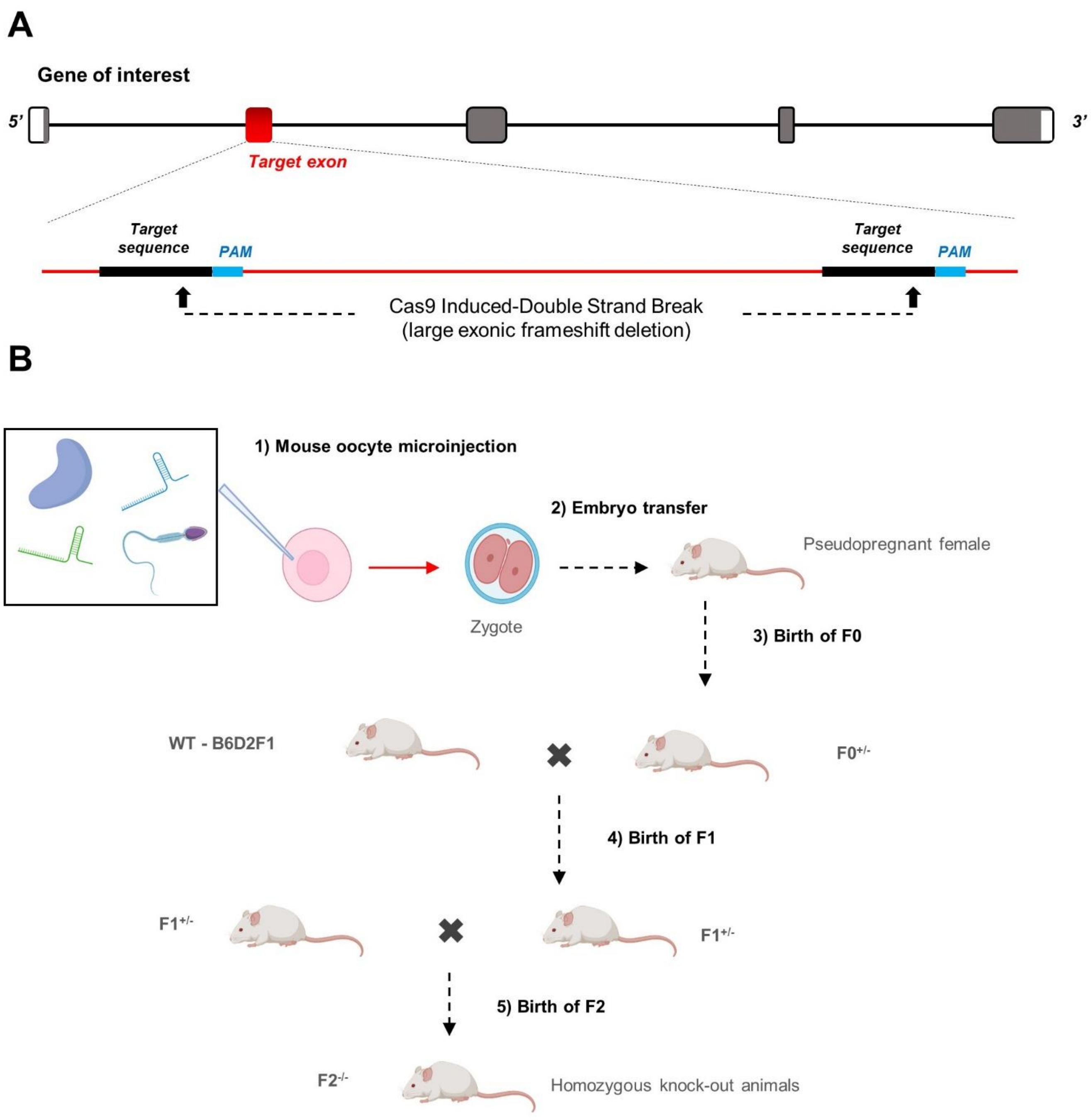

Supplementary Figure S1. Gene editing using CRISPR/Cas9 system to generate knockout (KO) mouse lines. **A)** Schematic presentation of a candidate gene for CRISPR/Cas9 editing. The exon in red was targeted with two gRNAs located in each exonic extremity. The Cas9 induces a double strand break (DSB) in each targeted region resulting in a large intra-exonic deletion. PAM: protospacer adjacent motif. **B)** To knock-out a candidate gene, the Cas9 and dual gRNAs were injected with a spermatozoa into the cytoplasm of a mature oocyte (ICSI). The obtained embryos were then transferred into a pseudopregnant female. The obtained pups (F0 generation) were genotyped and mated at adult age with B6D2F1 wild-type mice to produce F1 generation of pups. Heterozygous males and females carrying the same frameshift mutation were selected and mated to produce homozygous KO animals.

SUPPLEMENTARY MATERIALS

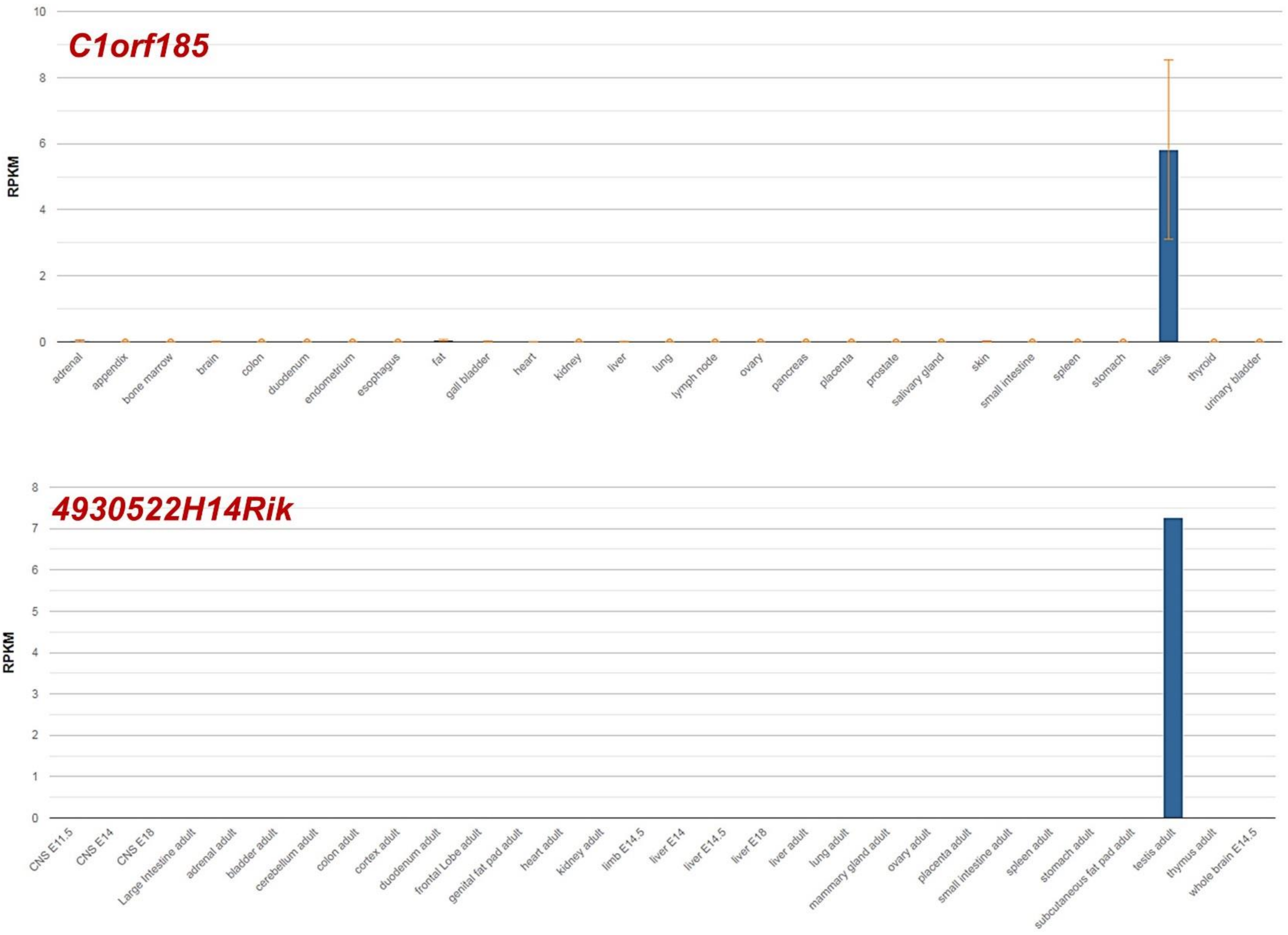

Supplementary Figure S2. Tissue expression pattern of *C1orf185* and its mouse ortgholog *493022H14Rik.* Graphical presentation of mRNA expression in different tissus including the gonades (testes and ovaries). We selected in NCBI, HPA (Human protein atlas) data from to present the expression in human and and ENCODE for mouse.

SUPPLEMENTARY MATERIALS

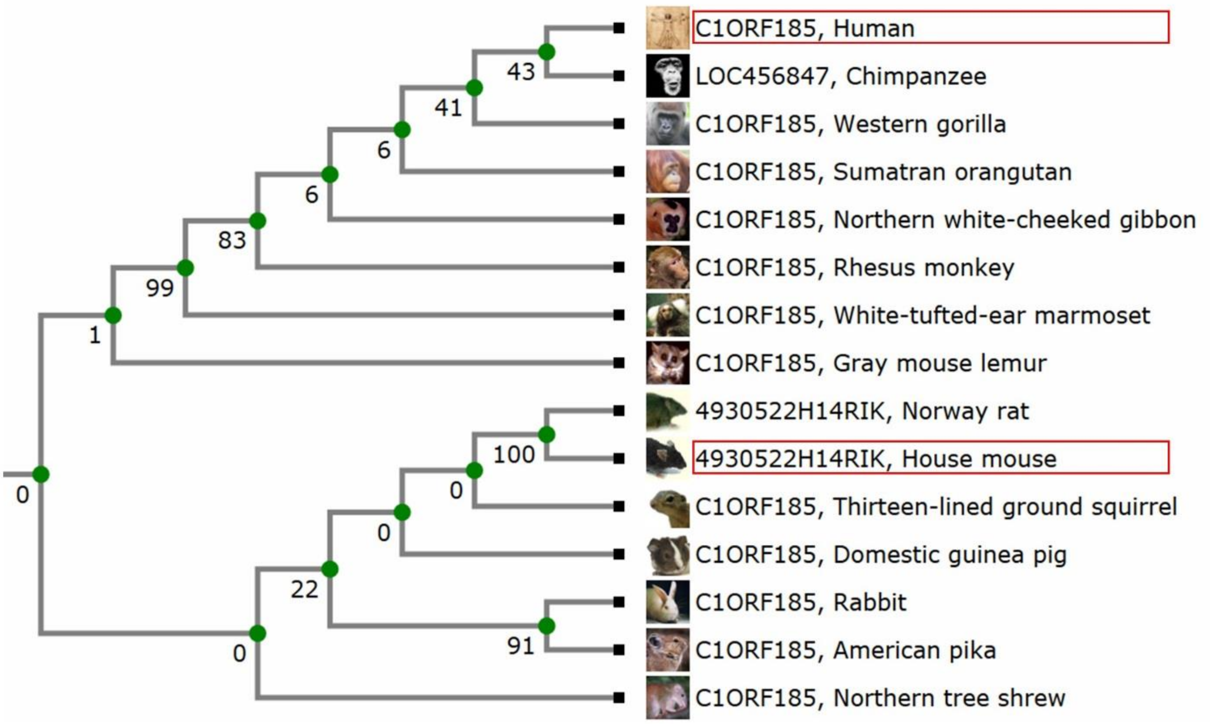

Supplementary Figure S3. **phylogenetic tree of** ***C1orf185 showing their orthologs and paralogs (if present).*** Graphical presentation generated from: http://www.treefam.org

SUPPLEMENTARY MATERIALS

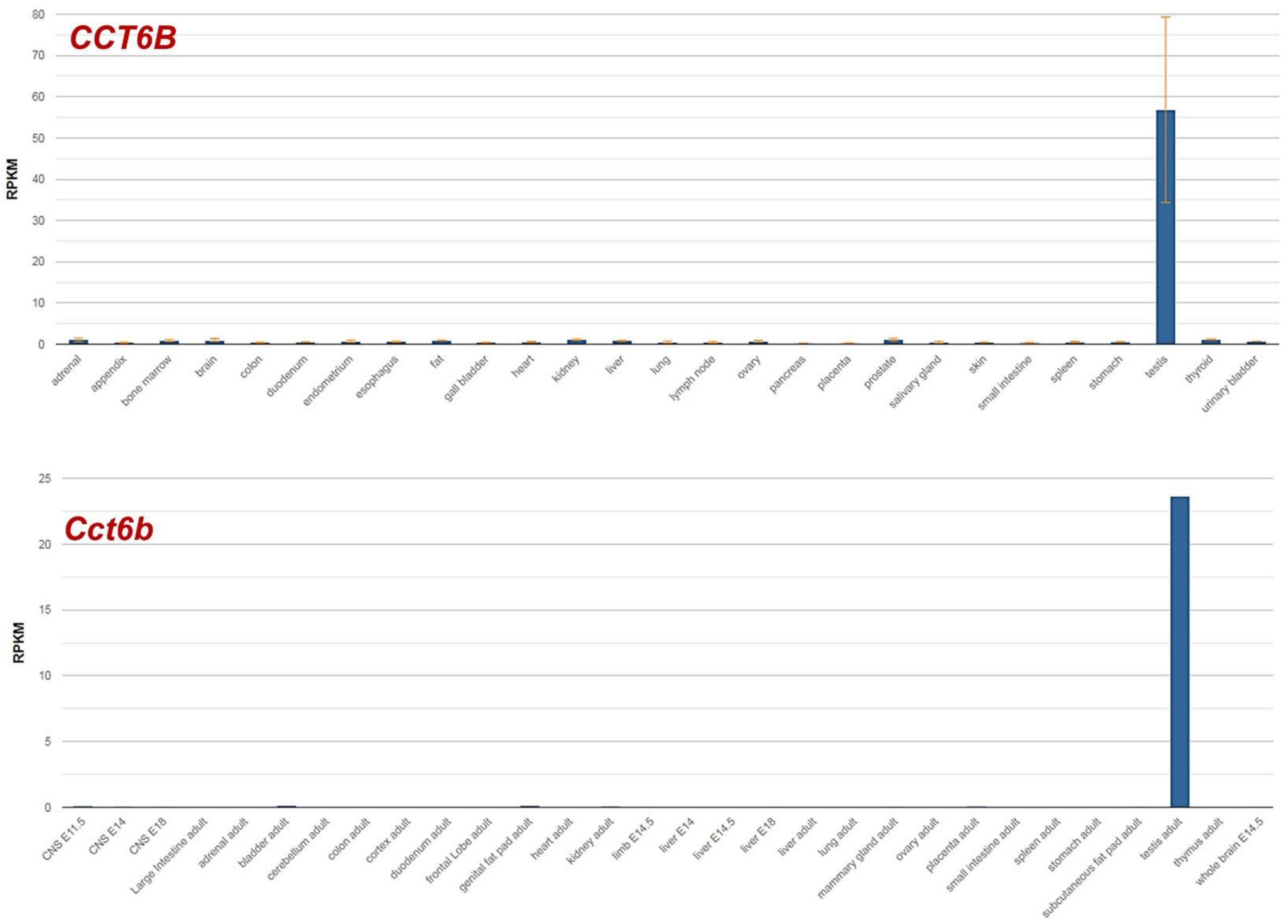

Supplementary Figure S4. Tissue expression pattern of *CCT6B and its mouse ortolog Cct6b.*
Graphical presentation of mRNA expression in different tissus including the gonades (testes and ovaries). We selected in NCBI, HPA (Human protein atlas) data from to present the expression in human and and ENCODE for mouse.

SUPPLEMENTARY MATERIALS

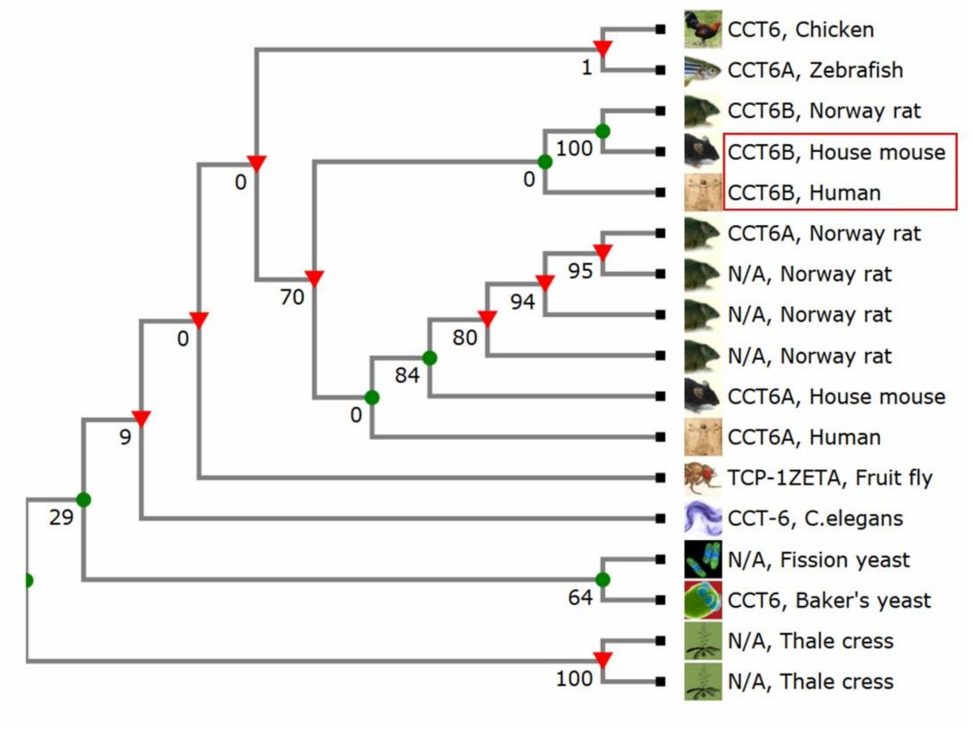

Supplementary Figure S5. phylogenetic tree of CCT6b ***showing their orthologs and paralogs (if present).***
Graphical presentation generated from: http://www.treefam.org
